# Atrial inflammation in different atrial fibrillation subtypes and its relation with clinical risk factors

**DOI:** 10.1007/s00392-020-01619-8

**Published:** 2020-02-18

**Authors:** Linghe Wu, R. W. Emmens, J. van Wezenbeek, W. Stooker, C. P. Allaart, A. B. A. Vonk, A. C. van Rossum, H. W. M. Niessen, P. A. J. Krijnen

**Affiliations:** 1 Department of Pathology, Amsterdam University Medical Centers, location VUmc and AMC, Room L2-114, Meibergdreef 9, 1105 AZ Amsterdam, The Netherlands; 2 Amsterdam Cardiovascular Sciences, Amsterdam, The Netherlands; 3 Department of Cardiac Surgery, Amsterdam UMC, location VU Medical Center, Amsterdam, The Netherlands; 4 Department of Cardiac Surgery, OLVG, Amsterdam, The Netherlands; 5 Department of Cardiology, Amsterdam UMC, location VU Medical Center, Amsterdam, The Netherlands

**Keywords:** Atrial fibrillation, Lymphocytes, Inflammation, Risk factors

## Abstract

**Objective:**

Inflammation of the atria is an important factor in the pathogenesis of atrial fibrillation (AF). Whether the extent of atrial inflammation relates with clinical risk factors of AF, however, is largely unknown. This we have studied comparing patients with paroxysmal and long-standing persistent/permanent AF.

**Methods:**

Left atrial tissue was obtained from 50 AF patients (paroxysmal = 20, long-standing persistent/permanent = 30) that underwent a left atrial ablation procedure either or not in combination with coronary artery bypass grafting and/or valve surgery. Herein, the numbers of CD45+ and CD3+ inflammatory cells were quantified and correlated with the AF risk factors age, gender, diabetes, and blood CRP levels.

**Results:**

The numbers of CD45+ and CD3+ cells were significantly higher in the adipose tissue of the atria compared with the myocardium in all AF patients but did not differ between AF subtypes. The numbers of CD45+ and CD3+ cells did not relate significantly to gender or diabetes in any of the AF subtypes. However, the inflammatory infiltrates as well as CK-MB and CRP blood levels increased significantly with increasing age in long-standing persistent/permanent AF and a moderate positive correlation was found between the extent of atrial inflammation and the CRP blood levels in both AF subtypes.

**Conclusion:**

The extent of left atrial inflammation in AF patients was not related to the AF risk factors, diabetes and gender, but was associated with increasing age in patients with long-standing persistent/permanent AF. This may be indicative for a role of inflammation in the progression to long-standing persistent/permanent AF with increasing age.

**Graphic abstract:**

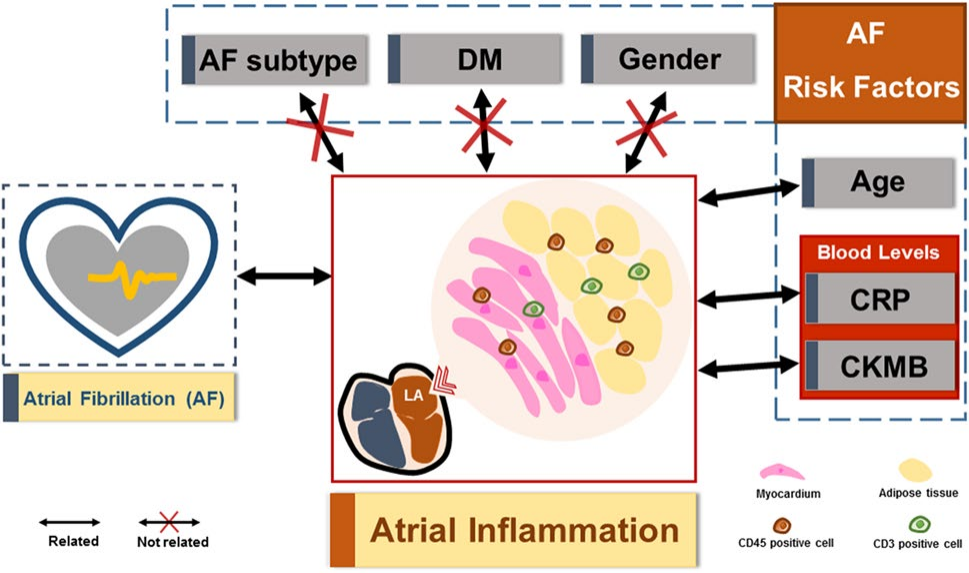

## Introduction

Atrial fibrillation (AF) is the most common form of sustained cardiac arrhythmia [1], and is associated with an increased risk of stroke, heart failure, and death [2–4]. Estimates on the incidence of AF (diagnosed and undiagnosed) in the general adult population ranged from 0.95 to 2.5% [5]. Multiple clinical risk factors are associated with a significantly increased prevalence of AF, including age [6], gender [5], and diabetes mellitus (DM) [3, 7], although the exact pathophysiology is still unknown.

Accumulating evidence suggests that inflammation is an important denominator in the pathogenesis of AF. For instance, increased systemic inflammation appears to relate to AF burden and persistence [8]. Markers of systemic inflammation such as C-reactive protein (CRP) blood levels were found to be increased in AF patients and did correlate positively with poor clinical outcome [8, 9]. In addition, increased infiltration of CD45+ leukocytes and CD3+ T lymphocytes has been observed in the atria of AF patients [10, 11], more in the adipose tissue than in the myocardium [11]. However, whether the extent of atrial inflammation relates to clinical risk factors of AF or systemic inflammation has been scarcely investigated. A recent study found no correlation between the number of atrial CD3+ T lymphocytes and CD68+ macrophages and age or diabetes [10] in patients with long-standing persistent AF, although this was studied in the atrial myocardium only.

Different AF subtypes are recognized, including paroxysmal AF (lasts < 7 days, self-terminating); persistent AF (lasts 7 days to 1 year, terminated with cardioversion); and the chronic forms long-standing persistent AF (lasts > 1 year, rhythm control therapy is still considered) and permanent AF [12]. Paroxysmal AF can progress over time to these chronic continuous AF subtypes [12]. The differences in circulating levels of IL-6, IL-10, TNF-α, and N-terminal-pro-brian-type natriuretic peptide (NTpBNP) found between paroxysmal and long-standing persistent/permanent AF [13–15] indicate that atrial inflammation may differ between AF subtypes also, although unknown is whether this coincides with a difference in atrial inflammation.

Therefore, we studied the relation between the inflammatory infiltrate in the left atrium with clinical risk factors of AF (age, gender, diabetes, and CRP blood levels), comparing paroxysmal and long-standing persistent/permanent AF.

## Materials and methods

### Patients

For this study, leftover tissue of the auricles of the left atrium of AF patients (*n* = 50) was used, that was obtained during cardiac surgery at the Onze Lieve Vrouwen Gasthuis hospital in Amsterdam. Based on AF subtype [12], we selected paroxysmal AF patients (*n* = 20) and long-standing persistent AF (*n* = 23) and permanent AF patients’ (*n* = 7). details of all included AF patients are listed in Table 1. As a control group (no AF), left atria tissue was obtained at autopsy from patients (*n* = 14) without any form of heart disease and without systemic infection at the department of Pathology at the VUmc. All autopsies were performed within 24 h after death and the bodies were stored and refrigerated. The causes of death are listed in Table 2. The atria tissue from AF patients was taken directly after having access to the atria, independent of the surgical procedure. After excision, the atrial tissue was immediately fixed in 4% formalin and subsequently embedded in paraffin for immunohistochemical analyses. Clinically determined pre-operative CRP blood levels were used for the analyses. The use of leftover patient materials for research after completion of the diagnostic process of post-mortem patients is conform patient contract in the VU Medical Center (VUmc) and this includes obtaining explicit written consent form relatives, in accordance with ethical guidelines set up by the World Medical Association (The declaration of Helsinki).

**Table 1:** Cardiac surgery of AF patients (*n* = 50)

	Age	Sex	AF subtype	Cardiac surgery	
		(M/F)		LA ablation	Concomitant cardiac surgery
1	58	M	Paroxysmal	Minimal invasive PVI + box	
2	76	M	Paroxysmal	Left atrial MAZE	Aortic valve replacement Mitral valve annuloplasty CABG
3	74	M	Paroxysmal	PVI via midsternotomy	CABG
4	69	F	Paroxysmal	PVI via midsternotomy	Aortic valve replacement
5	65	F	Paroxysmal	PVI via midsternotomy)	Aortic valve replacement
6	66	M	Paroxysmal	PVI via midsternotomy	CABG
7	63	F	Paroxysmal	PVI via midsternotomy	CABG
8	70	M	Paroxysmal	Left atrial MAZE	CABG
9	48	F	Paroxysmal	Minimal invasive PVI	
10	77	F	Paroxysmal	PVI via midsternotomy	Aortic valve replacement
11	66	F	Paroxysmal	Minimal invasive PVI	
12	76	M	Paroxysmal	Minimal invasive PVI	
13	55	M	Paroxysmal	Minimal invasive PVI	
14	74	M	Paroxysmal	Left auricle amputation	CABG
15	56	M	Paroxysmal	PVI via midsternotomy	CABG
16	65	M	Paroxysmal	PVI via midsternotomy	CABG
17	67	M	Paroxysmal	Minimal invasive PVI	
18	76	M	Paroxysmal	Left auricle amputation	CABG
19	79	F	Paroxysmal	Laft atrial MAZE	Aortic valve replacement Mitral valve annuloplasty
20	41	M	Paroxysmal	Minimal invasive PVI	
1	52	M	Long-standing persistent	PVI via midsternotomy	CABG
2	72	M	Long-standing persistent	Cox-Maze IV	Mitral valve annuloplasty Tricuspid valve annuloplasty
3	38	M	Long-standing persistent	Minimal invasive PVI + box	
4	55	M	Long-standing persistent	Minimal invasive PVI + box	
5	48	M	Long-standing persistent	Minimal invasive PVI + box	
6	60	F	Long-standing persistent	Left atrial MAZE	CABG
7	65	M	Long-standing persistent	Cox-Maze IV	CABG
8	58	M	Long-standing persistent	Minimal invasive PVI + box	
9	74	M	Permanent	Left auricle amputation	Mitral valve annuloplasty CABG
10	46	F	Long-standing persistent	Minimal invasive PVI + box	
11	69	F	Long-standing persistent	Minimal invasive PVI + box	
12	58	M	Long-standing persistent	Minimal invasive PVI + box	
13	67	M	Long-standing persistent	Minimal invasive PVI + box	
14	75	M	Long-standing persistent	Left auricle amputation	Aortic valve replacement CABG
15	56	M	Long-standing persistent	Minimal invasive PVI + box	
16	56	M	Long-standing persistent	Minimal invasive PVI + box	
17	67	M	Long-standing persistent	Minimal invasive PVI + box	
18	76	F	Long-standing persistent	Cox-Maze IV	Mitral valve annuloplasty Tricuspid valve annuloplasty CABG
19	78	M	Permanent	Left auricle amputation	Mitral valve annuloplasty Tricuspid valve annuloplasty CABG
20	80	M	Permanent	Left auricle amputation	CABG
21	50	M	Permanent	Left auricle amputation	Aortic valve replacement
22	54	M	Long-standing persistent	Minimal invasive PVI + box	
23	84	M	Permanent	Left auricle amputation	Aortic valve replacement CABG
24	73	M	Permanent	Left auricle amputation	CABG
25	40	M	Long-standing persistent	Minimal invasive PVI + box	
26	73	F	Permanent	Left auricle amputation	Mitral valve replacement Tricuspid valve annuloplasty CABG Left/right atrium reduction
27	50	F	Long-standing persistent	Minimal invasive PVI + box	
28	73	M	Long-standing persistent	Left atrial MAZE	Mitral valve annuloplasty Tricuspid valve annuloplasty
29	58	F	Long-standing persistent	Minimal invasive PVI + box	
30	66	M	Long-standing persistent	Minimal invasive PVI + box	

*AF* atrial fibrillation, *M* male, *F* female, *LA* left atrium, *PVI* pulmonary vein isolation, *CABG* coronary artery bypass grafting

**Table 2:** Characteristics of control patients (*n* = 14)

Patient	Cause of death
#1	Hypovolemic shock
#2	Interstitial fibrosis of the lungs and pneumonia
#3	Dissection of the aorta
#4	B-cell lymphoma of the brain
#5	Dissection of the aorta
#6	Unknown
#7	Dissection of the aorta
#8	Unknown
#9	Hemorrhage of the brain
#10	Anaphylactic shock
#11	Brain infarction
#12	Unknown
#13	Dissection of the aorta
#14	Hemorrhage of the brain

### Immunohistochemistry

Four-µm sections were deparaffinized and dehydrated prior to the immunohistochemical staining. To block endogenous peroxidase activity, the sections were incubated in 0.3% H_2_O_2_ in methanol for 30 min for staining with rabbit–anti-human CD3 (T lymphocytes; Dako Agilent, Amstelveen, The Netherlands), antigen retrieval was performed by boiling the sections in Tris–EDTA buffer (10 mM, pH 9.0) for 10 min. No antigen retrieval was used for staining with mouse anti-human CD45 (lymphocytes; Dako Agilent). The sections were washed with phosphate-buffered saline (PBS) and then, incubated with the primary antibodies against CD45 or CD3, both diluted 1:50 in normal antibody diluent (Dako Agilent) for 1 h at room temperature. Subsequently, the sections were washed with PBS and incubated with Envision HRP anti-mouse/rabbit for 30 min. For every immunohistochemical staining, a negative control (whereby the staining protocol was followed, but without incubation with the primary antibody) and a positive control (whereby the staining protocol was followed on tonsil tissue) were included. In all cases the negative control showed no staining and the positive control showed the appropriate staining (not shown). Two independent observers scored the tissue slides (R.W. Emmens and L. Wu), and the interobserver variation was < 10%.

### Quantification of inflammatory cells

In the present study, we have analyzed CD45+ lymphocytes that include B and T lymphocytes, and CD3+ T lymphocytes. Although CD45 (leukocyte common antigen) is present on non-lymphocytic cells also, it can be used as a general lymphocyte marker based on the morphology of positive-staining cells [16]. Only round cells with scant cytoplasm and a distinct peripheral reactivity for CD45 were counted. As a previous study showed that predominantly T rather than B lymphocytes infiltrated the atria of AF patients, we focused on T cells only [10].

All extravascular CD45+ and CD3+ cells were counted manually using a light microscope. The cells were quantified separately in the atrial myocardium and the atrial adipose tissue. Thereafter, the surface areas of the atria myocardial and adipose tissue were measured for each sample using Qprodit v3.2 (Leica Microsystems, Rijswijk, The Netherlands). The number of CD45+ and CD3+ cells/mm^2^ was then calculated.

### Statistical analysis

Statistical analysis was performed with SPSS (Windows version 2.0, IBM Corp, Armonk, NY), and figures were made by Prism software version 7 (GraphPad Software, La Jolla, CA, USA). Putative differences in patient characteristics and disease history between the groups were analyzed using the Fisher’s exact test. Putative differences in atrial inflammation between the groups were analyzed using either a Mann–Whitney *U* tests for asymmetrically distributed data or an independent *T* test for normally distributed data. While correlations were determined using the Pearson or Spearman’s rank correlation coefficient if it was not normal distributed. For overall comparisons of differences between more than two groups used, a one-way ANOVA was used for normally distributed and a Kruskal–Wallis test for asymmetrically distributed data. *p* values < 0.05 were considered statistically significant.

## Results

### Study cohort

The clinical characteristics of the AF (*n* = 50) patients that were included in this study are depicted in Table 3. A higher percentage of males (77%) were present in the long-standing persistent/permanent AF group than in the paroxysmal AF group (65%), while diabetes (20%), recent myocardial infarction (10%), angina pectoris (10%), and hypertension (5%) were more prevalent in the paroxysmal AF group compared with the long-standing persistent/permanent group (13%, 7%, 7% and 3%, respectively). However, none of these characteristics differed significantly between the paroxysmal and long-standing persistent/permanent AF groups.

**Table 3:** Patients characteristics (*n* = 50)

Measurement	Paroxysmal AF (*n* = 20)	LS-PE/PER AF (*n* = 30)	*p* value
Age, years (mean ± SD)	66 (± 10.2)	62 (± 12.2)	0.435
Male/female	13/7 (65%/35%)	23/7 (77%/23%)	0.522
Diabetes mellitus	4 (20%)	4 (13%)	0.679
Blood markers			
Leukocytes (× 109/L)	5.8 (± 3.6)	4.7 (± 3.2)	0.174
Highest CK-MB mass (μg/L)	28.2 (± 19.1)	34.7 (± 39.0)	0.879
CRP (mg/L)	5.3 (± 8.6)	12.2 (± 42.8)	0.337
Cardiovascular disease			
Recent myocardial infarction	2 (10%)	2 (7%)	> 0.999
Angina pectoris	2 (10%)	2 (7%)	> 0.999
Hypertension	1 (5%)	1 (3%)	> 0.999

*AF* atrial fibrillation, *LS-PE/PER AF* long-standing persistent and permanent AF, *CK-MB* creatinine kinase isoenzyme MB, *CRP* C-reactive protein

### Inflammatory cell infiltration in different AF subtypes

**Fig. 1:**
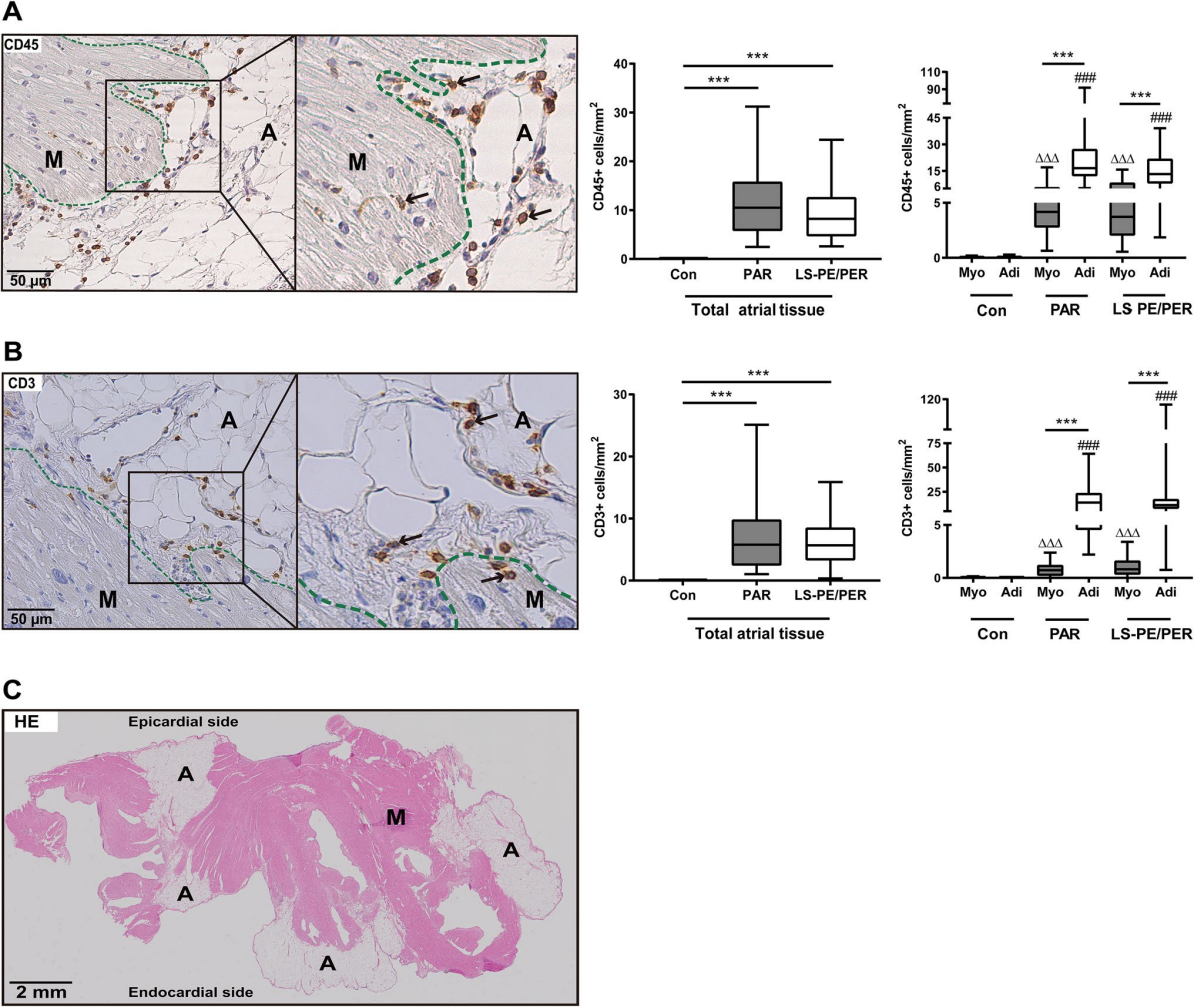
Inflammatory cells in the atria of patients with AF. An example of CD45+ (**a**) and CD3+ (**b**) cells (black arrows) in the atria of patients with AF. *M* myocardium, *A* adipose tissue, scale bar 50 µm. The number of CD45+ and CD3+ cells/mm^2^ in the myocardium (Myo) and adipose tissue (Adi) in the atria of control patients without AF (Con), patients with paroxysmal (PAR), long-standing persistent/permanent (LS-PE/PER) AF. (**c**) Hematoxylin–eosin stained cross section of the left atrial wall of an AF patient showing the spatial distribution of adipose tissue (A) and myocardium (M). Scar bar 2 mm. Data are presented as box plot with median and min–max percentiles (whiskers). Bars represent mean ± SD. ΔΔΔ means compared with myocardium of control group; ### means compared with adipose tissue of the control group. ***p < 0.001, ^ΔΔΔ^*p* < 0.001, ^###^*p* < 0.001

Extravascular CD45+ and CD3+ cells were observed in the left atrial appendages of AF patients, both in the myocardium and in the atrial adipose tissue (Fig. 1). These inflammatory cells did not predominantly co-localize with areas of fibrosis in the atria. In all AF patients, the numbers of CD45+ (Fig. 1a) and CD3+ (Fig. 1b) cells/mm^2^ present in the total left atrial tissue were significantly higher than in control patients (*p* < 0.001). The CD45+ and CD3+ cell densities did not differ significantly between paroxysmal AF patients and long-standing persistent/permanent AF patients. Previously, in paroxysmal AF patients, we found significantly more CD45+ cells in the adipose tissue of the atria compared with the myocardium [11]. Therefore, we also analyzed these two atrial layers separately. The adipose tissue was in majority located at the epicardial side, although infiltrations of adipose tissue into the myocardium up to the endocardium were also observed (Fig. 1c). The CD45+ and CD3+ cells were quantified in the adipose tissue regardless of location in the atria as a whole. Both in the atria of paroxysmal and long-standing persistent/permanent AF patients, the numbers of CD45+ and CD3+ cells/mm^2^ were significantly higher in the atrial adipose tissue compared with the myocardium. However, also here, no significant differences were found between the numbers of CD45+ and CD3+ cells/mm^2^ in these atrial layers between paroxysmal and long-standing persistent/permanent AF.

### The relation between clinical risk factors of AF and atrial inflammation

**Fig. 2:**
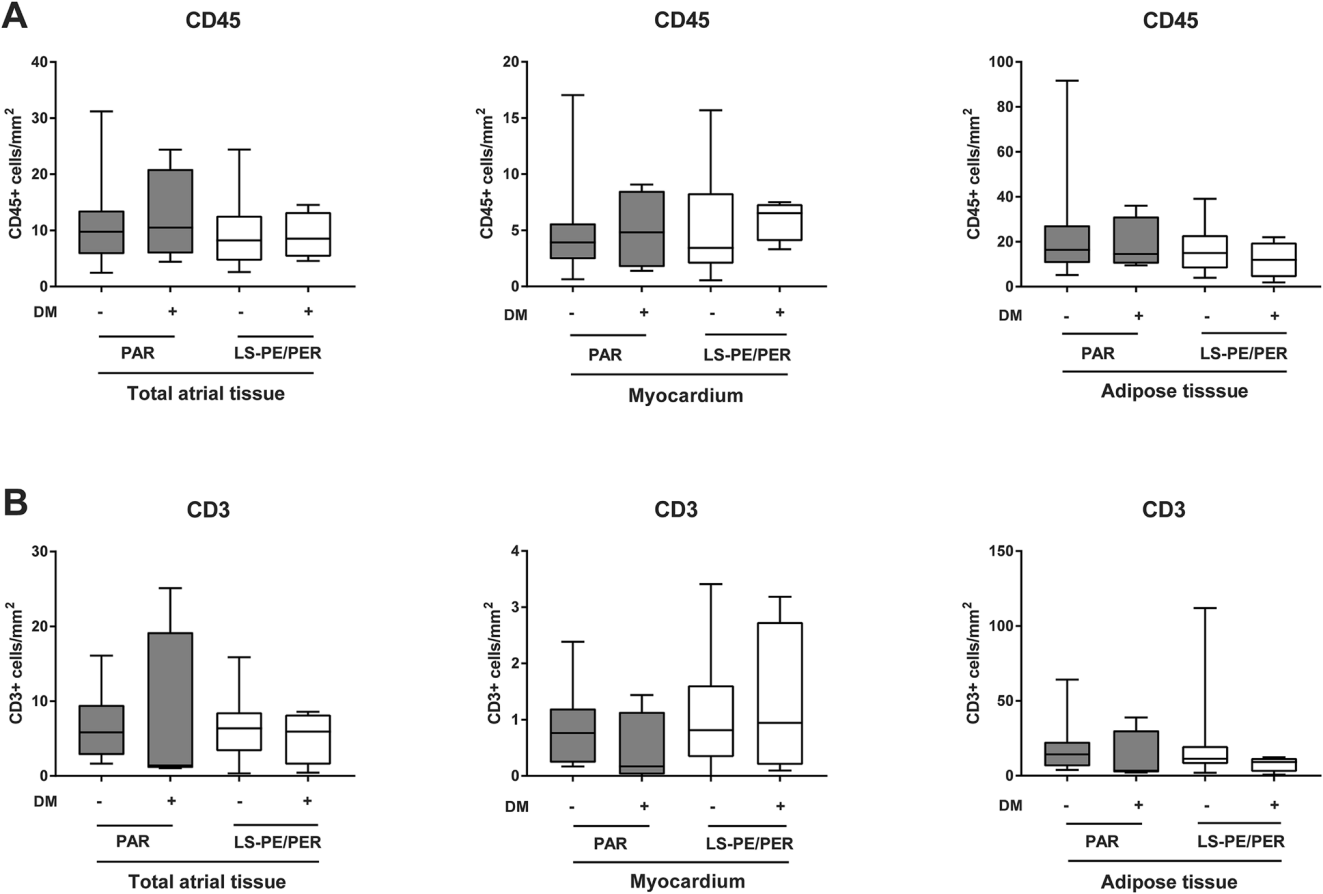
The atrial inflammatory cells infiltrate in diabetic versus non-diabetic AF patients. The number of CD45+ (**a**) and CD3+ (**b**) cells in the myocardium and adipose tissue in paroxysmal (PAR) and long-standing persistent/permanent (LS-PE/PER) AF patients with (*n* = 8) or without DM (*n* = 42). Data are presented as box plot with median and min–max percentiles (whiskers). Bars represent mean ± SD

**Fig. 3:**
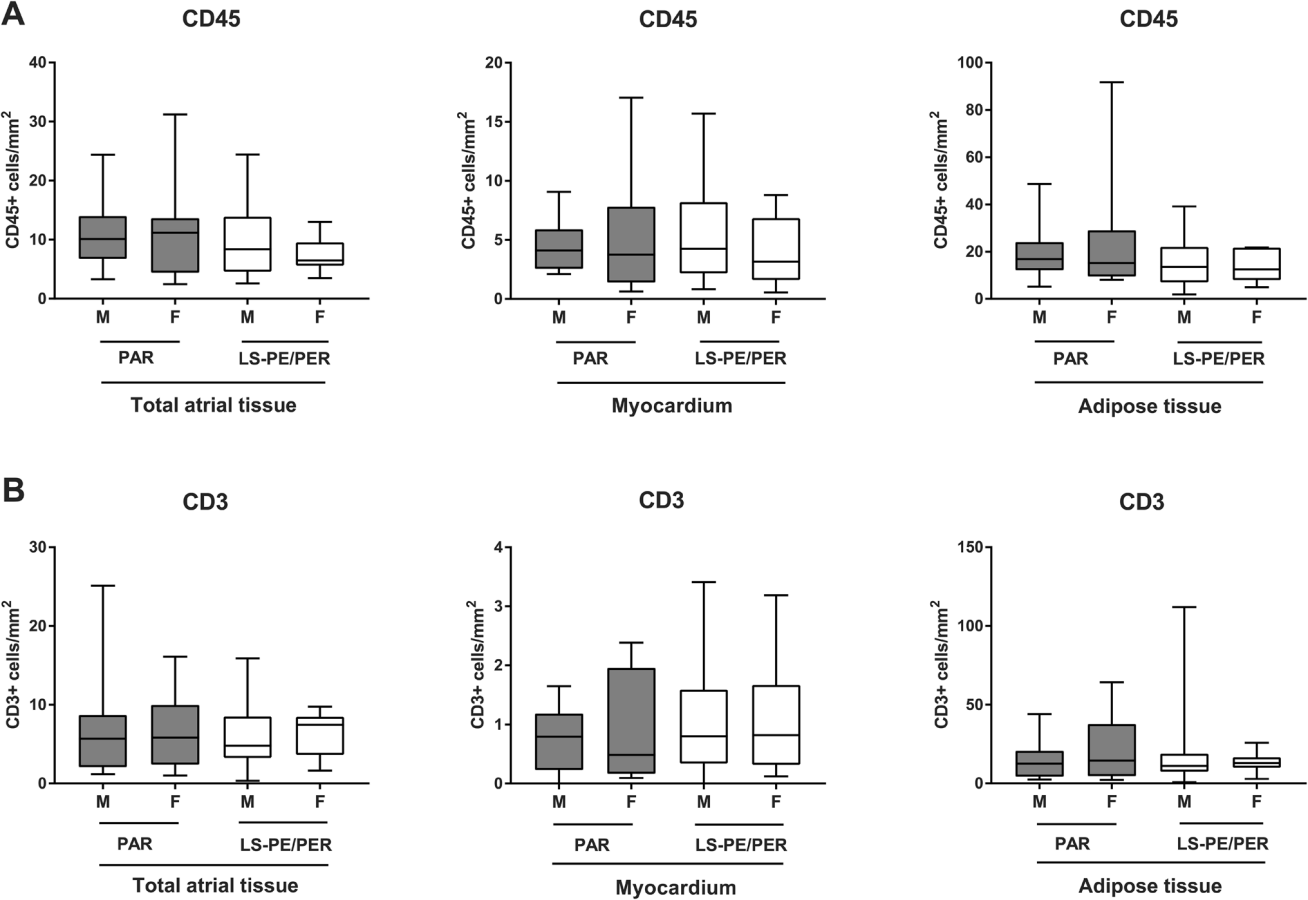
The atrial inflammatory cell infiltrate in male versus female AF patients. The number of CD45+ **a** cells in the myocardium and adipose tissue of male (*n* = 36) and female (*n* = 14) patients with paroxysmal (PAR) and long-standing persistent/permanent (LS-PE/PER) AF. The number of CD3+ **b** cells in the myocardium and adipose tissue of male (M) and female (F) patients with AF. Data are presented as box plot with median and min–max percentiles (whiskers). Bars represent mean ± SD

We subsequently correlated the clinical risk factor diabetes, gender, age, and CRP blood levels with the inflammatory cell density in the atria. No significant differences were found in the number of CD45+ (Fig. 2a) and CD3+ (Fig. 2b) cells/mm^2^ in the total atria tissue, nor in the myocardium and adipose tissue layers, between paroxysmal and long-standing persistent/permanent AF patients with and without diabetes. Similarly, no significant differences were found in the number CD45+ (Fig. 3a) and CD3+ (Fig. 3b) cells/mm^2^ between male and female AF patients in the total atrial tissue, nor in the myocardium and adipose tissue layers separately.

**Fig. 4:**
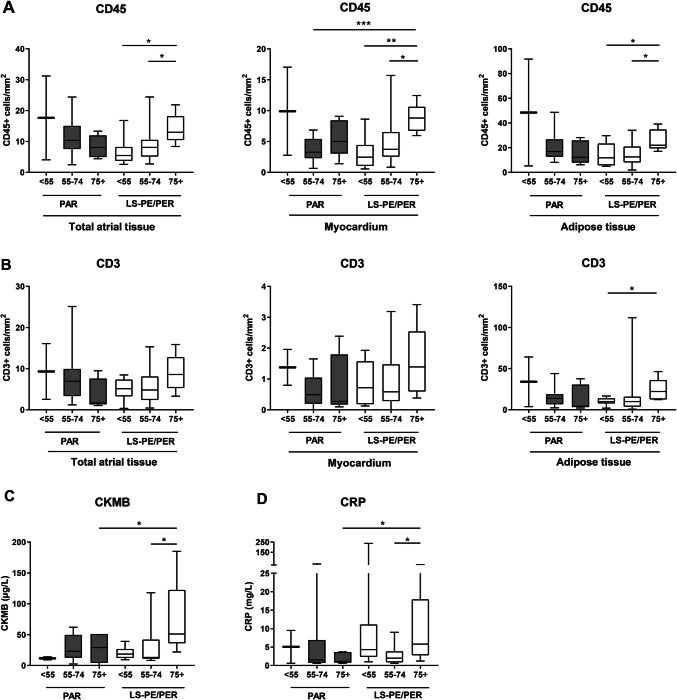
The atrial inflammatory cell infiltrate in AF patients of different age. The number of CD45+ **a** cells in the myocardium and adipose tissue in paroxysmal (PAR) and long-standing persistent/permanent (LS-PE/PER) AF patients aged below 55 years old (< 55, *n* = 10), between 55 and 74 years old (55–74, *n* = 30) and over 75 years old (75+, *n* = 10). The number of CD3+ **b** cells in the myocardium and adipose tissue of atria in AF patients. The blood levels of CK-MB (**c**) and CRP (**d**) in patients with paroxysmal (PAR) and long-standing persistent/permanent (LS-PE/PER) AF that were aged below 55 years (< 55), aged between 55 and 74 years (55–74) and aged over 75 years (75+). Data are presented as box plot with median and min–max percentiles (whiskers). Bars represent mean ± SD. *p < 0.05, **p < 0.01, ***p < 0.001

To analyze a putative effect of age, the inflammatory cell density in the atria was compared between patients below 55 years, between 55 and 74 years, and over 75 years. In long-standing persistent/permanent, but not paroxysmal AF patients, the number of CD45+ cells/mm^2^ in the total atrial tissue, as well as in the myocardium and adipose tissue layers, was significantly higher in patients of over 75 years old compared with patients below 55 years old and with patients between 55 and 74 years old (Fig. 4a). For the CD3+ cells/mm^2^, this was found only in the adipose tissue between patients over 75 years old and patients below 55 years old (Fig. 4b). Also the pre-operative CRP blood levels did not differ significantly between paroxysmal and long-standing persistent/permanent AF, but showed a moderate positive correlation with the CD3+ cell density in paroxysmal AF (*r* = 0.43, *p* = 0.058) and a moderate positive correlation with the CD45+ cell density in long-standing persistent/permanent AF (*r* = 0.38, *p* = 0.039). Moreover, we find an apparent association between the blood levels of both CK-MB and CRP, but not of leukocytes, and increasing age, specifically in patients with long-standing persistent/ permanent AF (LS-PE/PER), but not in paroxysmal AF patients (PAR). The CK-MB levels namely were significantly higher in patients of over 75 years old compared with patients below 55 years old and with patients between 55 and 74 years old (Fig. 4c). For CRP level, this was found only between patients over 75 years old and patients between 55 and 74 years old (Fig. 4d). However, correlation analysis revealed that there was no significant correlation between the CK-MB levels and the extent of atrial inflammation, nor in PAR and LS-PE/PER patients, and also no correlation between the CK-MB and CRP blood levels in the LS-PE/PER patients.

Lastly, we found no significant differences in atrial inflammation between patients that underwent left atrial ablation only and those who additionally underwent coronary artery bypass grafting and/or aortic valve replacement surgery.

## Discussion

Inflammation of the left atrium has been shown to play an important role in the pathogenesis of AF, albeit limited knowledge is available on the effects of clinical AF risk factors hereon and whether it differs between the different AF subtypes. We now found in both paroxysmal and long-standing persistent/permanent AF that the number of CD45+ and CD3+ cells was significantly higher in the adipose tissue compared with the myocardium, but that this did not differ between AF subtypes. Interestingly, the amount of atrial inflammation was associated with age in long-standing persistent/permanent AF patients and not in paroxysmal AF patients, while other risk factors did not have this differential effect.

Both in paroxysmal and long-standing persistent/permanent AF, inflammation was significantly more profound in the adipose tissue of the atria than in the myocardium. This corresponds well with the pro-inflammatory microvascular activation we observed previously in the left atrial adipose tissue of patients with paroxysmal AF [11]. Also, previous studies have shown increased inflammatory activity in epicardial atrial adipose tissue, using 18-fluorodeoxglucose (FDG)-positron emission tomography (PET) [17] and that the epicardial adipose tissue is a source of inflammatory mediators [18]. Our current data thus supports these studies that point to the epicardial atrial adipose tissue as a possibly important source of atrial inflammation in AF.

With regard to associations between the extent of atrial inflammation and the AF risk factors, we observed an age-associated increase in inflammatory cell density in the atrial myocardium and adipose tissue in patients with long-standing persistent/permanent AF. This is remarkable since studies show that the immune system is impaired especially in the elderly, which amongst others is due to a decline in the production and function of lymphoid cells; a process called age-related immunosenescence [19]. The fact that this trend was not seen in paroxysmal AF patients suggests that in addition to the previously observed differences in atrial morphology [20] and inflammatory blood markers [14], differences may exist in the cellular inflammatory responses between these two subtypes of AF. It may also suggest but not prove, why patients with high age have a higher risk of developing long-standing persistent and permanent AF. We found no associations between the extent of atrial inflammation and the AF risk factors gender and diabetes. The latter is in line with the study of Smorodinova N et al. who also found no correlation between the atrial inflammatory infiltrate and diabetes [10]. We did find a moderate positive correlation between the extent of atrial inflammation and pre-operative CRP blood levels, both in paroxysmal and long-standing persistent/permanent AF patients. Interestingly, we also observed significantly higher CRP and CK-MB levels in elderly patients with long-standing persistent/permanent AF. This indicates that the increased atrial inflammation in these patients may relate to increased systemic inflammation and/or myocardial damage, although this needs to be further established. It was shown previously that CRP blood levels are increased in AF patients and that they correlated positively with AF diameter and duration both in paroxysmal [21] and persistent and permanent AF [22]. Our results may indicate that systemic and local atrial inflammations are related in AF.

It is known that cardiac surgery such as coronary artery bypass grafting (CABG) induces systemic inflammation [23, 24], and that postoperative atrial fibrillation can be triggered by CABG [25] and aortic valve replacement surgery [26–28]. This suggests that cardiac surgery may induce atrial inflammation. However, since in our study the left atrial tissue samples were obtained in all cases at the start of the procedures, it is unlikely that either the ablation procedures or concomitant surgery affected the results.

In conclusion, our study shows that AF coincides with an increase of lymphocytes in the atria, especially in the adipose tissue. The extent of this atrial inflammation does not seem to be associated with gender and diabetes, but was found to be more pronounced with advanced age in long-standing persistent and permanent AF. This could point to, but does not prove a role of progression of AF tot long-standing persistent and permanent AF with increasing age.

### Study limitation

One limitation of this study is that we only studied a selection of risk factors, but not all known risk factors for AF. This was because either certain risk factors were present in too few of our patients to allow reliable statical analyses or information about certain risk factors was not available. This was also true regarding concomitant factors such as drug use and left ventricular function. These should be included in future analyzes. Another limitation was the use of left atrial tissue from deceased patients without AF as a control group. Left atrial tissue from living patients without AF could not be obtained for this study. However, we do not believe that their death impacted the extent of atrial inflammation in these patients.

### What is already known about this subject?

Inflammation has been implicated as an important factor in the pathogenesis of atrial fibrillation (AF). Multiple studies have indeed shown an increased presence of inflammatory cells in left atrial tissue of AF patients. However, whether the extent of atrial inflammation relates with clinical risk factors of AF or with AF duration is largely unknown.

### What does this study add?

1. We show that the CD45+ and CD3+ cell densities in the atria of all AF patients are significantly higher in the adipose tissue compared with the myocardium. Moreover, we observed no differences in the extent of atrial inflammation between patients with paroxysmal and long-standing persistent/permanent AF.
2. We also show that the extent of atrial inflammation was not related to the AF risk factors diabetes and gender, but correlated positively with age in long-standing persistent/permanent AF patients, and with CRP blood levels both in paroxysmal and long-standing persistent/permanent AF patients.

### How might this impact on clinical practice?

This study shows that the extent of atrial inflammation in AF patients appears to be affected by age and by systemic inflammation.
